# Characterization of Resistance to Cereal Cyst Nematode, Agronomic Performance, and End-Use Quality Parameters in Four Perennial Wheat-*Thinopyrum intermedium* Lines

**DOI:** 10.3389/fpls.2020.594197

**Published:** 2020-11-10

**Authors:** Lei Cui, Yongkang Ren, Yanming Zhang, Zhaohui Tang, Qing Guo, Yuqi Niu, Wenze Yan, Yu Sun, Hongjie Li

**Affiliations:** ^1^College of Agriculture, Shanxi Agricultural University, Taiyuan, China; ^2^The National Engineering Laboratory of Crop Molecular Breeding, Institute of Crop Sciences, Chinese Academy of Agricultural Sciences, Beijing, China; ^3^Key Laboratory of Molecular Cytogenetics and Genetic Breeding of Heilongjiang Province, College of Life Science and Technology, Harbin Normal University, Harbin, China

**Keywords:** perennial wheat, *Thinopyrum intermedium*, cereal cyst nematode, agronomic performance, end-use quality parameters, molecular cytogenetic analysis

## Abstract

Perennial wheat is considered to be a practical way to increase the flexibility and profitability of sustainable agricultural system, as it can be either a forage grass or a grain crop. Four perennial wheat lines SX12-480, SX12-787, SX12-1150, and SX12-1269 were developed from a series of interspecific crosses between common wheat (*Triticum aestivum*, 2n = 42) or durum wheat (*T. turgidum* var. *durum*, 2n = 28) and the intermediate wheatgrass (*Thinopyrum intermedium*, 2n = 42). These lines were characterized by the vigorous regrowth for at least 3 years. The one- and 2-year-old plants had higher grain yield potential than the 3-year-old perennial plants. The decline of grain yield was associated with plant age-related effects on yield components. The perennial wheat lines were all resistant to both *Heterodera avenae* and *H. filipjevi*, the two distinct cereal cyst nematode species that occur in China, except that line SX12-787 exhibited moderate resistance only to *H. avenae*. The dual-purpose perennial wheat lines were evaluated for quality values of both defoliated grass and harvested grains in the form of amino acid profile, mineral concentration, and contents of protein and fiber. Difference in the quality profile was observed between the perennial lines. These perennial lines had an overall improved quality levels over those of the perennial wheat control Montana-2 (*T. turgidum* × *Th. intermedium*) and the annual wheat cultivar Jinchun 9. The amplification profiles of the molecular markers provided molecular evidence for the introgression of alien chromatin. Genomic *in situ* hybridization detected 16, 14, 14, and 12 *Th. intermedium* chromosomes in lines SX12-480 (2n = 48), SX12-787 (2n = 56), SX12-1150 (2n = 56), and SX12-1269 (2n = 54), respectively, in addition to either 32 or the complete set of wheat chromosomes. The four perennial wheat-*Th. intermedium* lines described here provide valuable sources of perennial wheat for the dual-purpose application of both grain and forage.

## Introduction

Recent increase in the global attention has been paid to the development of perennial crop alternatives to annual crops (Jaikumar et al., 2012; Kantar et al., 2016; Cui et al., 2018). Enrichment of genetic diversity, soil conservation, improvement of water quality, and reduction in environmental pollution from fertilizers and pesticides are some of the benefits of perennial crops over annual cereals, especially on certain erodible and marginal lands (Bell et al., 2010). An obvious advantage of perennial wheat over annual cereal crops is its ability to produce forage for livestock and harvestable grain, similar to the way that other annual wheat is used to (Larkin et al., 2014). On the other hand, challenges exist in breeding perennial crops. One of the main issues is the potential buildup of disease pressure due to the persistence of perennial growth habit. Diseases, especially those caused by the soil-borne pathogens, could accumulate over years.

Perennial wheat is one of the promising perennial cereals for cold-temperate regions. It is developed by either direct domestication of wild perennial species or distant hybridization between annual wheat cultivars and perennial wild wheat relatives including *Thinopyrum* spp. (Murphy et al., 2009; Bell et al., 2010; Kantar et al., 2016; Cui et al., 2018). Because wild perennial species have beneficial characteristics, such as perennial growth habit, resistance to several pests and diseases, and nutritional benefits, they are useful in development of perennial wheat and improvement of the nutritional values of annual wheat through wide hybridization (Li and Wang, 2009; Mujoriya and Bodla, 2011; Hayes et al., 2012; Marti et al., 2015). Early attempts to develop perennial wheat can be dated back to the 1920s by Dr. N. V. Tsitin, former Soviet Union (Armstrong, 1936). Significant progress has been made in domesticating the perennial wheatgrass *Th. intermedium* (Host) Barkworth and D. R. Dewey leading to the development of cultivar Kernza as a dual-purpose forage and grain crop (Culman et al., 2013). Montana-2 (PI 505802) is the first commercial winter-hardy perennial wheat cultivar released in 1986; it was developed by crossing durum wheat [*T. turgidum* L. var. *durum* (Desf.); 2n = 28] with *Agrotriticum intermediodurum* Khizhnyak (syn. *Th. intermedium*) (Schulz-Schaeffer and Haller, 1987; Schulz-Schaeffer and Friebe, 1992).

Perennial wheat is desired to grow several years, so resistance to diseases, particularly those caused by certain soil-borne pathogens, is of particular importance. It has been shown that the wheat-*Thinopyrum* derivatives are highly resistant to various fungal and viral diseases, such as the rusts (caused by *Puccinia striiformis* Westend., *P. graminis* Pers.:Pers., and *P. triticina* Eriks.), Fusarium head blight (caused by *Fusarium graminearum* Schw.), powdery mildew (caused by *Blumeria graminis* DC. f. sp. *tritici* Marchal), eyespot (caused by *Tapesia yallundae* Wallwork & Spooner, syn. *Oculimacula yallundae* Wallwork & Spooner), *Barley Yellow Dwarf Virus* (BYDV), *Wheat Streak Mosaic Virus* (WSMV) and its vector wheat curl mite *Aceria tosichella* (Keifer) (Cox et al., 2002, 2005; Fedak and Han, 2005; Li et al., 2005; Jauhar et al., 2009; Li and Wang, 2009). However, little information is available on the potential of perennial wheatgrass as source of resistance against the soil-borne pathogens. Cereal cyst nematode (CCN, *Heterodera* spp.) is one of such parasites that causes significant yield losses in cereal crops and has become an important constraint on wheat production in many parts of the world (Peng et al., 2015; Smiley et al., 2017). This root parasitic nematode comprises several species (Nicol et al., 2011). *Heterodera avenae* Wollenweber is a dominant CCN species in China, which has been identified in the majority of wheat production regions, while *H. filipjevi* (Madzhidov) Stelter is an emerging threat to wheat production (Yuan et al., 2010; Peng et al., 2015). The nematode is able to complete a life cycle in one wheat cropping season. The eggs within the cysts can be viable in the soil for years until proper hatching conditions (Banyer and Fisher, 1971). Monoculture of susceptible wheat cultivars facilitates CCN reproduction in the soil, which ultimately results in outbreaks of the nematode. On the contrary, growing resistant cultivars not only suppresses the development and reproduction of CCN, but also limits the initial nematode density below the economic damage threshold for the next cropping seasons (Nicol et al., 2011; Cui et al., 2016). Extensive screenings in cultivated wheat germplasm have identified limited number of resistant cultivars and breeding lines (Xing et al., 2014; Peng et al., 2015; Cui et al., 2016). Many of the studies have focused on single CCN species only. Under natural field condition, mixed infestation can occur with the coexistence of *H. avenae* and *H. filipjevi*. Some of the known CCN resistant genes are not effective against both nematode species (Cui et al., 2020). Very recent evidence shows that resistance is mediated by distinct quantitative trait loci (QTL) depending on *Heterodera* species. Thus, sources conferring resistance to *H. filipjevi* can be susceptible upon *H. avenae* infestation and vice versa (Cui et al., 2020). Taken together, all of these complicate breeding efforts to develop CCN resistant wheat cultivars. Wild species *Thinopyrum* spp. has shown to be resistant to CCN. Wheat-*Thinopyrum* hybrids were reported to confer resistance to *H. filipjevi* (Li et al., 2012).

Another important challenge for developing perennial wheat is selection of cultivars with consistent grain production and equivalent grain yield to annual wheats since the hybrids with perennial parents have age-related decrease in grain yield and can produce 40–60% yield level relative to the annual counterpart (Bell et al., 2010; Jaikumar et al., 2012). In a perennial wheat breeding program conducted in Australia, several wheat-*Th. intermedium* lines were able to achieve approximately 75% of the grain yield of the annual wheat cultivar in the first year; however, these lines were hardly able to regrowth beyond the first harvest (Larkin et al., 2014). Perennial wheat lines showed persistent longevity for four consecutive years, but grain yield decreased dramatically. During the third and fourth years, these lines barely yielded harvestable grains. Modest grain production and yield decreases over ages might be due to competing sources in a perennial plant between grain yield and persistence, as well as the buildup of pathogens in the soil. For a dual-purpose perennial wheat cultivar, grain yield merely 40% relative to the annual wheat is considered to be profitable as perennial wheat provides forage for livestock (Bell et al., 2010). To understand the trade-off between plant longevity, forage biomass, and grain yield potential, it requires in-depth studies over multiple ages and years.

We developed four wheat-*Th. intermedium* lines that displayed strong perennial growth habit. These perennial lines were assessed for their field responses to CCN and their agronomical performances on consistency of yield components over multiple years. We measured the parameters relating to the nutritional components of one-year old defoliated grass and grains in comparison with the commercial annual wheat cultivar. The objectives of this study were to characterize their phenotypic responses to the two CCN species, agronomic performances, and quality properties in these perennial wheat lines. The chromosome compositions of these perennial wheat lines were analyzed by genome-specific markers and genomic *in situ* hybridization (GISH). To breed for the dual-purpose perennial wheat, emphasizes on consistent grain yield potential and improved forage biomass can offset inherent physiological constraints on grain production to a profitable and sustainable extent. Simultaneously, constantly high degree of resistance to soil-borne diseases is foremost objective to breed for superior perennial wheat.

## Materials and Methods

### Plant Materials

**FIGURE 1:**
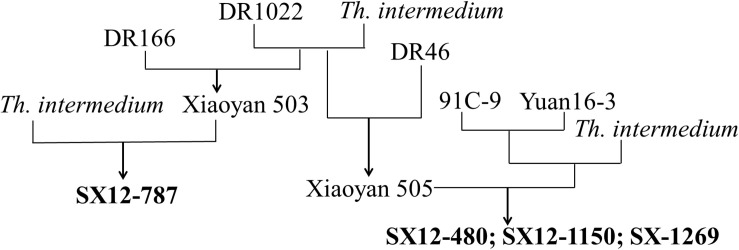
Pedigrees of the four perennial wheat lines SX12-480, SX12-787, SX12-1150, and SX12-1269.

The pedigrees of the four perennial wheat-*Th. intermedium* lines are shown in Figure 1. Line SX12-787 was produced by crossing hexaploid partial amphiploid Xiaoyan 503 (2n = 42, pedigree DR1022/*Th. intermedium*//DR116) with a *Th. intermedium* accession. Lines SX12-480, SX12-1150, and SX12-1269 were developed from the same cross between an octoploid partial amphiploid line (2n = 56, pedigree 91C-9/Yuan 16-3//*Th. intermedium*) and a hexaploid partial amphiploid Xiaoyan 505 (2n = 42, pedigree DR1022/*Th. intermedium*//DR46). The *Th. intermedium* accession and the durum wheat DR46, DR116, and DR1022 were provided by the Institute of Crop Sciences, Chinese Academy of Agricultural Sciences, Beijing, China. The Chinese wheat cultivars Wenmai 19 (Lankao 4/Wen 2540) and Jinchun 9 (Taigu male sterile line/Jinyan 163-12), and perennial wheat cultivar Montana-2 (MT-2, PI 505802) from the United States were used as the controls in the assessments of CCN resistance and quality parameters, respectively. An accession of *Th. intermedium* (2n = 42, JJJ^*s*^J^*s*^StSt) and common wheat cultivar Chinese Spring (2n = 42, AABBDD) were used to extract DNA for preparation of the probe or the blocker in the GISH analysis.

The perennial wheat lines were developed and assessed on the sandy loam soil at Dongyang Agricultural Experimental Station, Shanxi Agricultural University, Jinzhong, Shanxi province (37.56°N, 112.68°E, 801 m als). The local weather condition belongs to the temperate continental arid climate with an annual precipitation of 418 – 483 mm. The long-term annual mean temperature is 9.8°C, the mean frost-free period is 158 days, and the average temperature in winter ranges from −12°C to 12°C (China Meteorological Data Service Center, 2020)^1^.

### Field Evaluation of CCN Resistance

During the 2013–2014 and 2014–2015 growing seasons, reactions to *H. avenae* (pathotype Ha43) (Yuan et al., 2010) and *H. filipjevi* (pathotype Hfc-1) (Fu et al., 2011) were evaluated at Zhengzhou (ZZ, 34.44°N, 113.24°E, 171 m als) and Xuchang (XC, 34.04°N, 113.74°E, 98 m als), Henan province, respectively. A randomized complete block design with three replicates was used to arrange each line. In each year 20 selfing seeds of the second-year-old perennial plants from the growing season of 2012–2013 were sown in 2 m rows, 0.3 m apart. Eight to ten plants per line were randomly sampled with soil cylinders from each plot at the Zadoks growth stage (GS) 77 (late grain-filling stage) (Zadoks et al., 1974). The roots were carefully raised with tap water in order to visually count the white females (cysts). The phenotype of each line was determined by averaging number of white females (cysts) on the roots per plant and separated into five categories: resistant (<5), moderately resistant (5–10), moderately susceptible (11–14), and susceptible (15–25), and highly susceptible (>25) (Nicol et al., 2009).

### Evaluation of Agronomic Traits

Perennial wheat lines were grown in plots consisting of 3 m-long row and spaced 0.4 m that were arranged in a randomized complete block design with three replicates. Twenty-five seeds were sown in a row in early October 2011. Defoliation was performed twice during June (both grain and forage harvest) and November (forage cut preparing for over-wintering) each year, afterwards regrow ability was observed. The agronomic performances were investigated in three consecutive years. Depending on the agronomic traits, evaluation was performed before the first grain and forage harvest at June, or immediately after harvesting. The “first-year plant” refers to individuals during their first growing season (2011–2012), the “second-year plant” denotes plants that show persistent regrowth during the second growing season (2012–2013), and the “third-year plant” represents plants displaying post-harvest regrowth habit for three consecutive years during the third growing season (2013–2014). From each line, plant height (cm), spike length (cm), and number of spikelets per spike were recorded in ten randomly sampled individuals from each plot during the growing seasons of 2011–2012, 2012–2013, and 2013–2014. Plant height was determined from the ground level to the top of the spike, and spike length was measured from the base of rachis to the top of the spike. In addition, number of spikelets per spike was enumerated and the spikes were threshed in a bench micro-thresher to determine thousand-kernel weight (g). The common practices of irrigation (twice, one before over-winter stage immediately after defoliation, and the other after grain and forage harvesting at June), fertilizers (nitrogen, phosphorus and potassium), and pesticides were conducted following the local farm system.

### Analysis of End-Use Quality

The end-use quality of the perennial wheat lines, including 16 amino acids, crude fat, crude protein, crude fiber, ash, phosphorus, iron, selenium and carotene were analyzed by the Inspection and Testing Center for Quality of Cereals and Their Products (Harbin), Ministry of Agriculture and Rural Affairs, China. Forage of the one-year old plants for each line was defoliated and samples were dried at 70°C for 48 h. Forage dry matter (300 g per sample) was taken for quality analysis. Grains (300 g, dry weight) from the first-year plants and common wheat cultivar Jinmai 9 were collected for quality measurements. Method for determination of amino acid profile in forage dry matter was described in the National Standard (GB/T) 18246-2000 (Standardization Administration of the People’s Republic of China, 2000). Measurements of crude protein, crude fat, and crude fiber in forage dry matter were based on the National Standards (GB/T) 6432-1994, 6433-2006, 6434-2006, respectively (Standardization Administration of the People’s Republic of China, 1994, 2006a, b). Measurements of phosphorus, iron, carotene, and selenium concentrations were performed as described in the National Standards (GB, GB/T) GB/T 6437-2002, GB/T 13885-2003, GB/T 5009.83-2003, and GB 5009.93-2010, respectively (Standardization Administration of the People’s Republic of China, 2002, 2003a, b, 2010). To determine the quality levels in the dried grains, methods used to assess the amino acid composition, crude protein, and ash contents were described in the National Standards (GB/T) GB/T 5009. 124-2003 (Standardization Administration of the People’s Republic of China, 2003c), the Agricultural Industry Standard (NY/T) 3-1982 (Ministry of Agriculture and Rural Affairs of the People’s Republic of China, 1982), and GB/T 5505-2008 (Standardization Administration of the People’s Republic of China, 2008), respectively.

### Molecular Marker Analysis

Primer pairs for the three simple sequence repeat (SSR) markers (*Xedm 28*, *Xedm16*, and *Xedm105*), one sequence characterized amplified region (SCAR) marker (*SCAR1248*), and one marker obtained from repetitive DNA sequence from *Th. elongatum* (*2P1*/*2P2*) were used to characterize alien chromatin in perennial wheat lines (Table 1) (Wang and Wei, 1995; You et al., 2002; Li et al., 2005; Mullan et al., 2005; Hu et al., 2012). Leaves of four perennial wheat lines, *Th. intermedium*, wheat cultivar Chinese Spring were harvested for DNA isolation using a cetyltrimethyl ammonium bromide (CTAB) based protocol (Fulton et al., 1995). Amplification of the SSR and the SCAR markers was conducted in a 25 μL reaction including 2 μL genomic DNA (10–30 ng/μL), 2.5 μL of PCR buffer (10×), 2.5 μL of dNTP, 1 μL of forward primer and reverse primer each, 15.7 μL deionized water, and 0.3 μL of RT-*Taq*. Cycling conditions consisted of incubation at 94°C for 5 min, 40 cycles of 94°C for 30 s, annealing temperature varied depending on different primers for 45 s, and 72°C for 1 min, and 72°C for 10 min (Yan et al., 2020). DNA amplification was carried out in an Eppendorf 9700 thermocycler (Eppendorf, Hamburg, Germany). The amplification of DNA using the primer pair 2P1/2P2 was conducted in a 20 μL reaction mixture that contained 2 μL 100 ng/μL genomic DNA, 1 μL 10 μmol/L of each primer, 10 μL 2 × *Taq* PCR Master Mix (Taingen Biotech, Beijing, China), and 6 μL sterilized ddH_2_O. The profile of DNA amplification was initial denaturation at 94°C for 3 min, followed by 35 cycles of 1 min at 94°C, 47–52°C ramp annealing for 45 s, 1 min for extension at 72°C, and a final extension step at 72°C for 10 min (Li et al., 2005). Separation and visualization of PCR products for SSR markers were completed as described by Zhao et al. (2013). Amplified products of markers SCAR1248 and 2P1/2P2 were electrophoretically separated on a 1.5% agarose gel, stained with ethidium bromide and observed under an ultraviolet (UV) light.

**TABLE 1:** Molecular markers used for characterization of the alien chromatin in the perennial wheat lines.

Marker	Genome/chromosome target	Primer (5′-3′)	Annealing temperature	PCR product size	Source of primers
SSR-*Xedm28*	*Thinopyrum elongatum* chromosomes 2ES and 3EL	F-GCTCACTCACGCATCATAGC	53.7	151 bp	You et al., 2002; Mullan et al., 2005
		R-GTTGGCGGAATCCTTCTTC			
SSR-*Xedm105*	*Th. elongatum* chromosome 7EL	F-ACCGCCAGGGAGCTCTGC	57	237 bp	You et al., 2002; Mullan et al., 2005
		R-GATGTCCTTCTGGCCGTACT			
SSR-*Xedm16*	*Th. elongatum* chromosome 7ES	F-TCACCTAACAGCACCACGAG	55.8	165 bp	Mullan et al., 2005
		R-GCCGAGTACCAGCAGTACCA			
*SCAR1248*	*Pseudoroegneria strigosa* St genome	F-TTAGACATCATGAGCACACC	55	850 bp	Hu et al., 2012
		R-ATGATGCAGCAGCAAATTACA			
2P1/2P2	*Thinopyrum* genus	F-ACAATCTGAAAATCTGGACA	47–52 ramp annealing	277 bp	Wang and Wei, 1995; Li et al., 2005
		R-TCATATTGAGACTCCTATAA			

### Genomic *in situ* Hybridization (GISH)

Seeds of the perennial wheat lines were germinated in Petri dishes at 23°C for 24 h, 4°C for 48 h and 23°C for 27.5 h. Root tips 1–1.5 cm in length were pretreated in ice water (0–4°C) for 24 h, and then fixed in ethanol-acetic acid (3:1) for 24 h at room temperature. The fixed root tips were squashed in 45% acetic acid after staining with 1% acetocarmine for 2 h. Chromosome numbers were enumerated under a light microscope (Leica DM LS2, Mannheim, Germany). Genomic DNA of *Th. intermedium* extracted using a CTAB method was labeled with DIG-Nick-Translation mix (Roche, Mannheim, Germany) as a probe, and sheared genomic DNA of Chinese Spring was used as a blocker. Antidigoxin Rhodamine and 4,6-diamidino-2-phenylindole (Roche, Mannheim, Germany) were loaded on slides prior to dark incubation. Hybridization signals were visualized under a Leica fluorescence microscope (Leica DM6000B, Mannheim, Germany). Photos were taken by a Leica digital camera (Model DFC480). The detailed GISH protocol was described by Yan et al. (2020).

### Statistical Analysis

Analysis of variance (ANOVA) for the phenotypic responses to two CCN species was performed considering genotype, year, CCN species, genotype by CCN species, genotype by year, year by CCN species, and genotype by year by CCN species interactions as the main factors. The ANOVA for the agronomic traits was performed comprising genotype, year, and genotype by year interaction as the main factors. The ANOVA for forage and grain quality characteristics was performed considering genotype as the main factor. The Fisher’s Least Significance Difference (LSD) was used to perform multiple comparisons for differentiating the significant difference among genotypes and among plant ages (i.e., the first, second, and third-year plants) on the means of each parameters examined. Statistical analysis was performed in the IBM SPSS Statistics for Windows, Version 26.0 (IBM Corp., Armonk, NY, United States).

## Results

### Development of the Perennial Wheats

**FIGURE 2:**
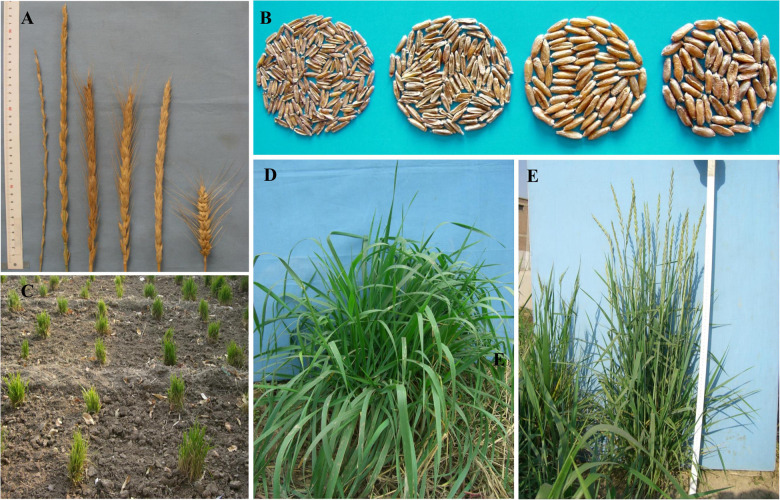
Agro-morphological traits of the perennial lines. **(A)** Morphology segregation in spikes, from left to right *Th. intermedium* spike, four distinct progeny spikes in the F_2_ and F_3_ generations (wheatgrass type and three different intermediate types), and spike of common wheat cultivar. **(B)** Morphology segregation of seeds in the F_2_ and F_3_ generations, *Th. intermedium* (first-left) and three different types of progeny seeds (wheatgrass type and followed by two intermediate types). **(C)** Vegetative growth state after winter in April. **(D)** Regenerated vegetative growth after defoliation. **(E)** Performance at the maturity in the field.

In order to develop perennial wheats for feed and food consumption, hundreds of interspecific crosses were made in the early 2000. During the early generations from F_1_ to F_3_, the targets of selection focused on the traits associated with perennial growth habit, such as non-shattering heads, high fertility, and ability of regrowth in the fields. The F_1_ hybrids showed a vigorous regrowth habit and were often sterile. Some F_1_ plants were able to persist adequately even for more than 7 years in the field and developed numerous tillers, but only a few tillers produced viable seeds. Then, the F_2_ and F_3_ plants were obtained by either self-pollination or backcrossing to common wheat. However, the persisting life cycle was converted into annual growth habit in some F_2_ and F_3_ plants. In parallel, the F_2_ plants with perennial growth habit were obtained via backcrossing to hexaploid partial amphiploid lines or *Th. intermedium*. Importantly, the fertility of the perennial plants was restored. A wide range of variation in the major agronomic traits, such as spike and seed morphology, was observed at the early generations (Figures 2A,B). Both spike and seed morphology in the F_2_ and F_3_ plants segregated for the wheat, intermediate, and wheatgrass types (Figures 2A,B). The intermediate and/or wheatgrass types of F_2_ and F_3_ plants, which exhibited regrow habit with high seed-setting rates, were selected for further selection for flowering time, plant height and agronomic performances in later generations. A total of 78 lines were obtained at the F_6_ generation. Some of these lines showed perennial habit under favorable greenhouse conditions, but did not regrow when transplanted to the field, possibly due to winter killing. Whereas, a number of hybrid plants survived after experience of the hot and dry summer and harsh cold winter at the farm in Shanxi province (37.56°N, 112.68°E, 801 m als). Among them, lines SX12-480, SX12-787, SX12-1150, and SX12-1269 were able to regrow from the crowns of plants after forage removal (Figures 2C,D) and remained their productivity for at least 3 years (Figure 2E). They were selected as the perennial wheat lines for further characterization of the agronomic performances, quality characteristics, and disease resilience.

### Field Resistance to CCN

The ANOVA based on the general linear (GLM) model was carried out for the number of cysts on roots of the perennial lines and the control wheat cultivar Wenmai 19 challenged by *H. avenae* and *H. filipjevi* under natural field infestation during the two growing seasons 2013–2014 and 2014–2015 (Table 2). The results indicated that phenotypic responses were independent of year but depended on the genotype and CCN species. The interaction effects were absent, suggesting the high heritability and consistency of the resistance.

**TABLE 2:** Mean squares of the analysis of variance for the phenotypic responses to *Heterodera avenae* and *H. filipjevi* of the perennial wheat lines and the control common wheat cultivar Wenmai 19 during the growing seasons of 2013–2014 and 2014–2015.

Source of variation	df	Number of cysts
Genotype	4	633.95***
Year	1	10.24
CCN species	1	221.78**
Genotype × Year	3	40.59
Genotype × CCN species	4	29.83
Year × CCN species	1	2.94
Genotype × Year × CCN species	3	33.89

**P < 0.01; ***P < 0.001.

**FIGURE 3:**
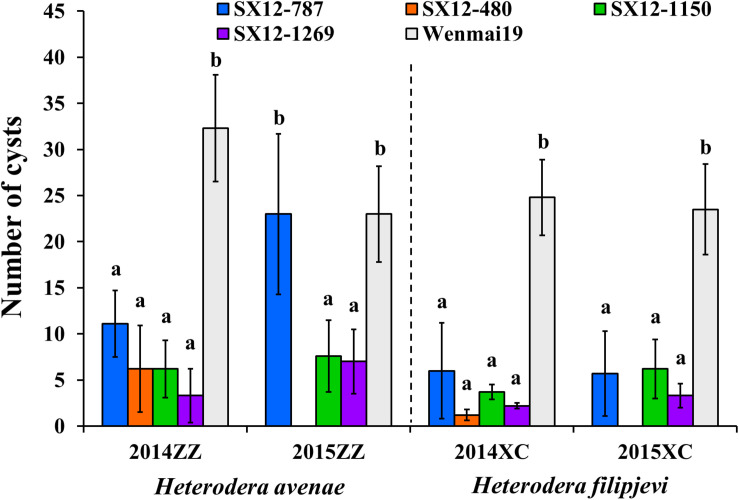
Phenotypic responses of perennial wheat lines SX12-480, SX12-787, SX12-1150, and SX12-1269 and the annual wheat cultivar Wenmai 19 to *Heterodera avenae* (ZZ) and *H. filipjevi* (XC) in the field of Zhengzhou (ZZ) and Xuchang (XC), respectively, based on the number of white females (cysts) during the two growing seasons 2013–2014 and 2014–2015. Bars with the same letters are not significantly different at *P* < 0.05.

At the field of Zhengzhou (ZZ), *H. avenae* is the main CCN species. In the growing season 2013–2014, the perennial wheat lines were highly resistant (line SX12-1269) or moderately resistant (lines SX12-480 and SX12-1150) to *H. avenae*. Although line SX12-787 displayed moderate level of susceptibility, the number of cysts (11.1 ± 3.6) was significantly lower than the susceptible control, an annual wheat cultivar Wenmai 19 (32.3 ± 5.8) (*P* < 0.05) (Figure 3). The field trial was repeated using the new seeds during the growing season 2014–2015 and line SX12-480 was excluded due to the very low germination rate. Lines SX12-1269 and SX12-1150 conferred moderate resistance to *H. avenae*, whereas line SX12-787 supported comparable number of cysts (23 ± 8.7) relative to that of susceptible control (23 ± 5.2) (Figure 3).

Upon natural infestation with *H. filipjevi* in the field of Xuchang (XC) in 2013–2014, lines SX12-480, SX12-1150, and SX12-1269 were able to mediate high levels of resistance and had significantly large impact on reducing the number of female cysts. Line SX12–787 also conferred resistance to *H. filipjevi*, but to a smaller extent (Figure 3). In the following year, the whole experiment was repeated using new seeds, and all the tested perennial lines were able to combat against *H. filipjevi*. In this case, SX12-1269 was highly resistant, and lines SX12-787 and SX12-1150 conferred a moderate level of resistance (Figure 3). Taken together, line SX12-1269 conferred high level of resistance against the two distinct CCN species in the field infestations, and the other lines were also able to produce significantly fewer white females on the roots than the susceptible wheat control Wenmai 19 (*P* < 0.05).

### Evaluation of Agronomic Traits and Grain Yield Components

**FIGURE 4:**
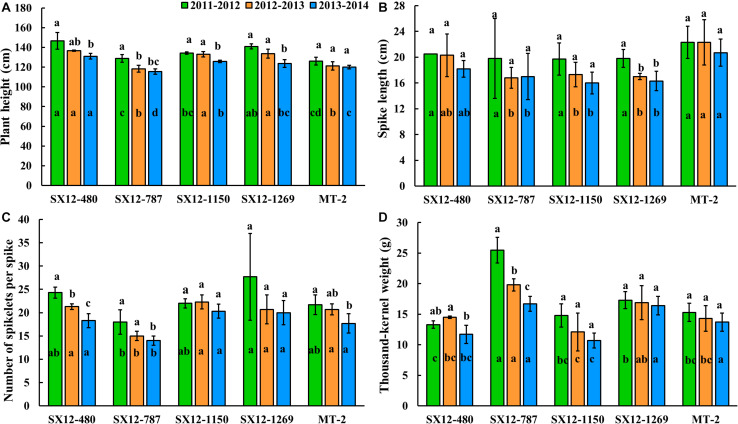
Assessment of agronomic traits including **(A)** plant height and **(B)** spike length and yield components including **(C)** number of spikes per spike and **(D)** thousand-kernel weight of perennial wheat lines developed from present study and the control perennial cultivar Montana-2 over the three growing seasons 2011–2012, 2012–2013, and 2013–2014 in Shanxi province, China. For each factor per growing season, different letters inside bars indicate a significant different among lines using the Fisher’s LSD (*P* < 0.05). For the tested variables of each line, different letters above bars represent difference among growing seasons (*P* < 0.05).

The ANOVA based on the GLM was carried out for plant height, spike length, number of spikelets per spike, and thousand-kernel weight of the perennial lines and the control perennial wheat cultivar MT-2 during the three consecutive years from 2011 to 2014 (Table 3 and Figure 4). Genotype and year were the determining factors of the differences in the four agronomic traits. The interaction effect of genotype by year was absent in plant height, spike length, and number of spikelets per spike, highlighting the high heritability of these traits. However, the significant difference in thousand-kernel weight was due to the factor of genotype by year interaction (Table 3). Lines SX12-480 and SX12-1269 (133.7–146.7 cm for plant height) were significantly taller than the other lines and the control MT-2 (*P* < 0.05) (Figure 4A). There was no significant difference on spike length among the four lines and MT-2 in their first growing season. In the second and third years, MT-2 and SX12-480 bore longer spikes (Figure 4B). Compared to MT-2, the four perennial lines produced comparable number of spikelets per spike across all the three growing seasons. Although not significant, improved number of spikelets per spike were found in lines SX12-1269, SX12-480, and SX12-1150 (Figure 4C). Furthermore, line SX12-787 stood out at all the growing seasons for the significantly higher thousand-kernel weight than those of the other lines (*P* < 0.05) (Figure 4D).

**TABLE 3:** Mean squares of the analysis of variance for agronomic traits and grain yield components of perennial wheat lines and the control perennial wheat cultivar Montana-2 during three growing seasons of 2011–2012, 2012–2013, and 2013–2014 representing the one-year-old, 2-year-old, and 3-year-old plants.

Source of variation	df	Plant height	Spike length	Number of spikelets per spike	Thousand-kernel weight
		cm	cm		g
Genotype	4	474.47***	28.81**	68.26***	99.29***
Year	2	556.63***	29.91*	82.47***	43.69***
Genotype × Year	8	24.7	1.82	7.02	9.62**

*P < 0.05; **P < 0.01; ***P < 0.001.

In parallel, to understand the grain yield consistency of perennial lines, a multi-year field study was conducted to compare the agronomic performance of the one-year old plants to the 2- and 3-year old ones (Figure 4). Comparing to the first-year plants, the 2-year old plants generally showed congruous plant height. However, the 3-year old plants of four perennial lines reduced their height to a significant extent except MT-2 (Figure 4A). Such a trend was also observed for number of spikelets per spike and thousand-kernel weight. Spikelet number and thousand-kernel weight were comparable over 2 years in all the lines tested. When plant life span extended, spikelet number and thousand-kernel weight declined remarkably. It is noteworthy that lines SX12-1150 and SX12-1269 maintained their productivity over plant age by producing comparable number of spikelets and thousand-kernel weight (Figures 4C,D). Generally, the perennial wheat lines displayed a reduction in yield potential with an effect of plant age. Climate variability over years might also have consequences on yield potential. It was difficult to separate the effects of weather fluctuations over years from the age of the plants.

### Assessments of Forage and Grain Quality-Related Characteristics of the Perennial Wheat Lines

**FIGURE 5:**
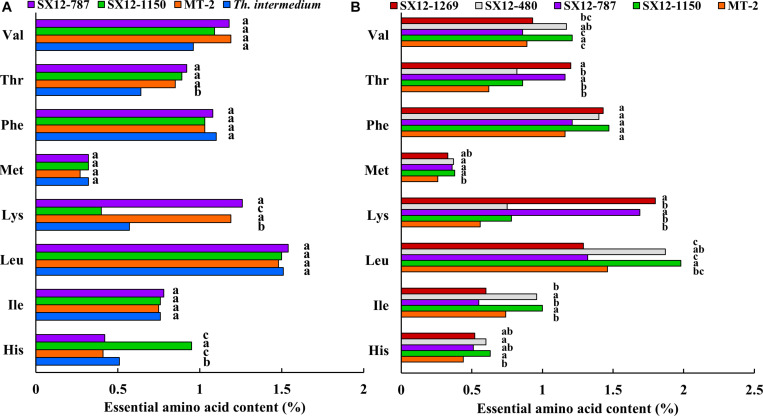
Essential amino acid content of forage and grain from perennial wheat lines and wheatgrass *Th. intermedium* during the growing season 2011–2012. Different letters next to the bars indicate a significant difference using the Fisher’s LSD (*P* < 0.05). Amino acid 3-letter codes, Val, valine; Leu, leucine; Ile, isoleucine; Phe, phenvlalanine; His, histidine; Lys, lysine; Met, methionine; Thr, threonine. **(A)** Essential amino acid content of forage. **(B)** Essential amino acid content of grain.

The first-year perennial lines, intermediate wheatgrass and an annual wheat cultivar Jinchun 9 were evaluated in the form of crude protein, crude fat, crude fiber content, the amino acid profile and the micronutrient contents (Tables 4, 5 and Figure 5).

**TABLE 4:** Nutritive and quality characteristics of forage and grain from perennial wheat lines, *Thinopyrum intermedium* and annual wheat cultivar Jinchun 9 during the growing season 2011–2012.

Quality trait	Forage				Grain					
	*Th. intermedium*	MT-2	SX12-1150	SX12-787	Jinchun 9	MT-2	SX12-1150	SX12-787	SX12-480	SX12-1269
Crude protein (%)	–	19.53^a^	18.93^a^	19.34^a^	12.90^b^	21.03^a^	25.21^a^	21.73^a^	25.22^a^	22.37^a^
Crude fat (%)	–	3.70^ab^	3.76^b^	2.78^a^	1.60^b^	2.81^a^	3.17^a^	3.03^a^	3.06^a^	2.90^a^
Crude fiber (%)	–	21.88^a^	20.88^a^	21.09^a^	–	–	–	–	–	–
Phosphorus	–	0.18%^a^	0.2%^ab^	0.24%^b^	2410.00 mg/kg^*c*^	–	3037.5 mg/kg a	3063.2 mg/kg a	3211.8 mg/kg b	3102.00 mg/kg^a^
Iron (mg/kg)	137.00^a^	223.00^b^	241.00^b^	151.00^a^	–	59.00^ab^	59.00^ab^	63.00^ab^	64.00^b^	48.00^a^
Selenium (mg/kg)	0.71^b^	0.34^a^	0.37^a^	0.40^a^	0.26^a^	0.27^a^	0.33^ab^	0.36^ab^	0.38^b^	0.31^ ab^
Carotene (mg/100 g)	–	8.98^a^	16.85^b^	9.85^a^	–	–	–	–	–	–

*–, missing value. Different letters in a row within each factor (forage and grain) indicate a significant difference using the Fisher’s LSD (P < 0.05).*

**TABLE 5:** Non-essential amino acid profile of forage and grains derived from the perennial wheat lines, the control MT-2, and *Thinopyrum intermedium*.

Amino acid (%)	Forage				Grain				
	*Th. intermedium*	MT-2	SX12-1150	SX12-787	MT-2	SX12-1150	SX12-787	SX12-480	SX12-1269
Alanine	0.75^a^	1.11^b^	1.19^b^	1.24^b^	0.70^a^	0.98^b^	0.8^ab^	0.94^b^	0.85^ab^
Arginine	0.95^a^	0.98^a^	1.33^b^	1.03^a^	0.84^a^	1.30^b^	1.11^a^	1.28^b^	1.22^b^
Aspartic acid	0.92^a^	1.77^b^	1.66^b^	1.85^b^	0.91^a^	1.37^b^	1.22^ab^	1.30^b^	1.09^ab^
Cystine	0.54^*d*^	0.00^a^	0.27^*c*^	0.21^b^	0.44^a^	0.52^a^	0.50^a^	0.55^a^	0.45^a^
Glutamic acid	6.94 b	2.58^a^	2.41^a^	2.43^a^	6.22^a^	8.76^b^	7.39^ab^	8.25^ab^	8.29^ab^
Glycine	0.79^a^	0.92^a^	0.92^a^	0.96^a^	0.73^a^	1.06^b^	0.91^ab^	1.00^b^	0.88^ab^
Proline	2.47 c	1.65^b^	1.21^a^	1.78^b^	2.49^a^	2.94^a^	2.23^a^	2.79^a^	2.73^a^
Serine	1.05^b^	0.75^a^	0.82^a^	0.85^a^	0.98^b^	1.37^*c*^	0.74^ab^	1.30^*c*^	0.67^a^
Tyrosine	0.42^a^	0.39^a^	0.37^a^	0.42^a^	0.56^a^	0.50^a^	0.58^a^	0.54^a^	0.62^a^

*Different letters in a row within each factor (forage and grain) indicate a significant difference using the Fisher’s LSD (P < 0.05).*

Forage harvested from the perennial lines had comparable concentrations of crude protein (96.9–99%), crude fat (75.1–101.6%), and crude fiber (95.4–96.4%) with those obtained from the commercial perennial wheat cultivar MT-2 (Table 4). The levels of crude protein and crude fat in grains varied tremendously between the perennial plants and those produced from the annual wheat cultivar Jinchun 9. These values in the perennial grains were 168–196% and 181–198% higher than those in Jinchun 9. Variability was found within the perennial genotypes. Although not significant, improved levels of both crude protein and crude fat comparing with MT-2 were found in all four perennial wheat lines.

Micronutrient concentrations for phosphorus (P) and carotene were found to be higher in the defoliated grass of perennial wheat lines SX12-1150 and SX12-787 than those produced from MT-2. Iron (Fe) level was enormously improved in the perennial lines compared to their perennial donor, *Th. intermedium*. Oppositely, forage obtained from *Th. intermedium* was a better source of selenium (Se). The concentrations of P and Se in the perennial grains were significantly richer than in the annual grains (*P <* 0.05). Among the perennial lines, SX12-480 was particularly intriguing due to high P and Se concentrations in grains and was significantly more than those in MT-2 (Table 4). Variation in Fe content was found among the perennial lines. Although not significant, high Fe concentration was observed in lines SX12-787 and SX12-480.

The essential amino acid contents of forage remained generally consistent among the perennial genotypes and wheatgrass, with only a few detectable differences (Figure 5A). Line SX12-1150 was characterized by significantly higher levels in histidine, but lower in lysine than that of the other tested lines (*P <* 0.05). Line SX12-787 was slightly lower in histidine and had significantly higher level in lysine. Wheatgrass was a great source of valine, phenylalanine and leucine (Figure 5A). When it came to the amino acid profile in grains, the perennial wheat lines were generally richer in comparison with those in MT-2. The increase in amino acid content ranged from 115.9 to 143.2% in histidine, 129.7 to 135.1% in isoleucine, 128.1 to 135.6% in leucine, 133.9 to 321.4% in lysine, 126.9 to 131.6% in methionine, 104.3 to 126.7% in phenylalanine, 132.3 to 193.5% in threonine, and 96.6 to 135.9% in valine (Figure 5B). There was a great variation found among the lines examined. Line SX12-1150 stood out for the highest contents in six out of eight tested essential amino acids including histidine, isoleucine, leucine, methionine, phenylalanine, and valine. Line SX12-787 was significantly higher in lysine and threonine than the control MT-2 (*P* < 0.05), whereas isoleucine content was the lowest. Line SX12-1269 had the highest level of lysine and threonine, but leucine level was found to be the lowest. Line SX12-480 ranked in the middle grades in the essential amino acid profile among the perennial lines (Figure 5B).

Furthermore, the non-essential amino acid profile was determined in both forage and grains obtained from the perennial lines and compared with the control MT-2 and *Th. intermedium* (Table 5). The intermediate wheatgrass had a great potential to the improvement of nutritional values, particularly the concentrations of cystine, glutamic acid, proline, and serine in the forage (Table 5). Line SX12-1150 had significantly higher amounts of arginine and cystine in the defoliated grass than those produced from the control MT-2 (*P* < 0.05). Moreover, grains harvested from SX12-1150 were the best sources of alanine, arginine, aspartic acid, glutamic acid, glycine, proline, and serine, which covers majority, if not all, of the non-essential amino acids (Table 5).

### Molecular and Cytogenetic and Characterization of the Perennial Wheat Lines

**FIGURE 6:**
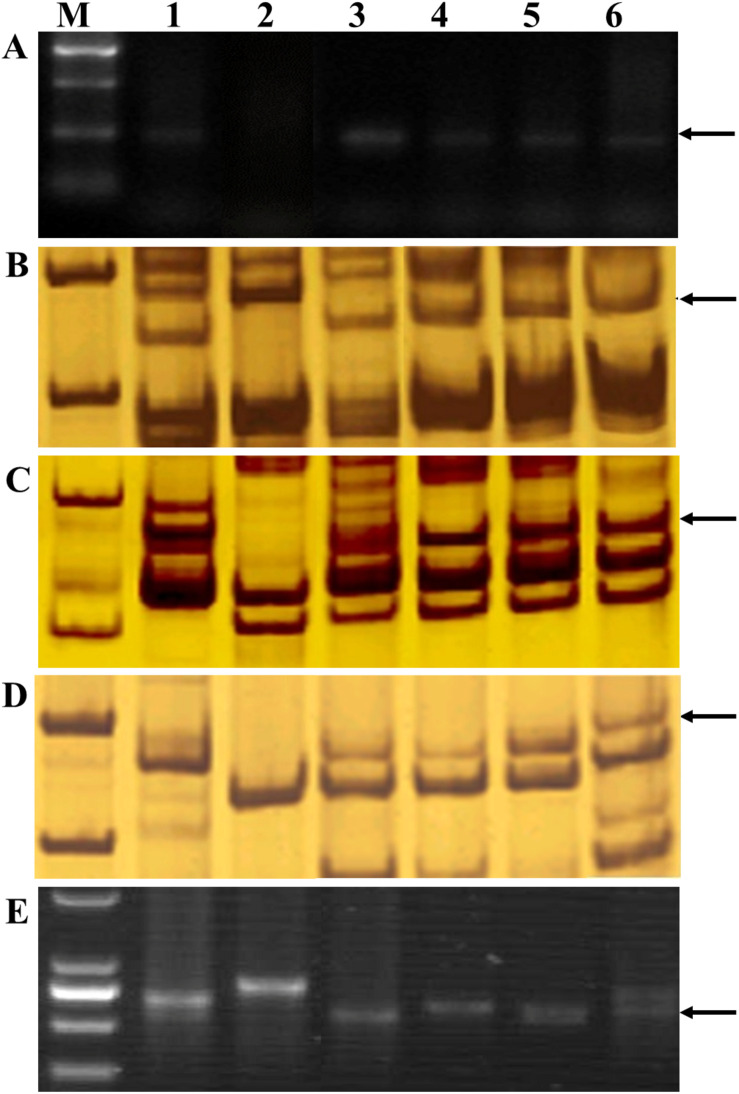
PCR amplification of alien chromosome specific markers **(A)** 2P1/2P2 (repetitive DNA sequence from *Th. elongatum*), **(B)** SSR-*Xedm28* (chromosomes 2ES and 3EL), **(C)** SSR-*Xedm16* (chromosome 7ES), **(D)** SSR-*Xedm105* (chromosome 7EL), and **(E)** *SCAR-1248* (St-genome specific marker). Lanes M, DNA size ladder; 1, *Th. intermedium*; 2, wheat cultivar Chinese Spring; 3, SX12-1150; 4, SX12-480; 5, SX12-1269; 6, SX12-787. Arrows indicate the diagnostic amplification products.

The parental genotype *Th. intermedium*, common wheat cultivar Chinese Spring, and the four perennial wheat lines SX12-480, SX12-787, SX12-1150, and SX12-1269 were genotyped using the E and St genome-specific molecular markers. The perennial wheat lines produced the similar diagnostic band of 277-bp in size with universal E genome marker 2P1/2P2 as their *Th. intermedium* parent, confirming the presence of the wheatgrass chromatin in their genomes. The common wheat cultivar Jinchun 9 did not amplify any of the fragments, indicating the absence of wheatgrass chromatin (Figure 6A). All the four perennial wheat lines produced an E genome–specific amplicon of 151-bp in size with marker *Xedm28* (located on 2ES and 3EL) (Figure 6B). Besides, all the four lines had chromosome 7E identified by 7ES and 7EL genome–specific amplification of markers *Xedm16* and *Xedm105*, which amplified the expected 165- and 237-bp bands, respectively (Figures 6C,D). Marker *SCAR-1248* produced an St genome–specific amplicon of 850-bp in the four lines, indicating that these materials contained the St-genome chromatins (Figure 6E).

**FIGURE 7:**
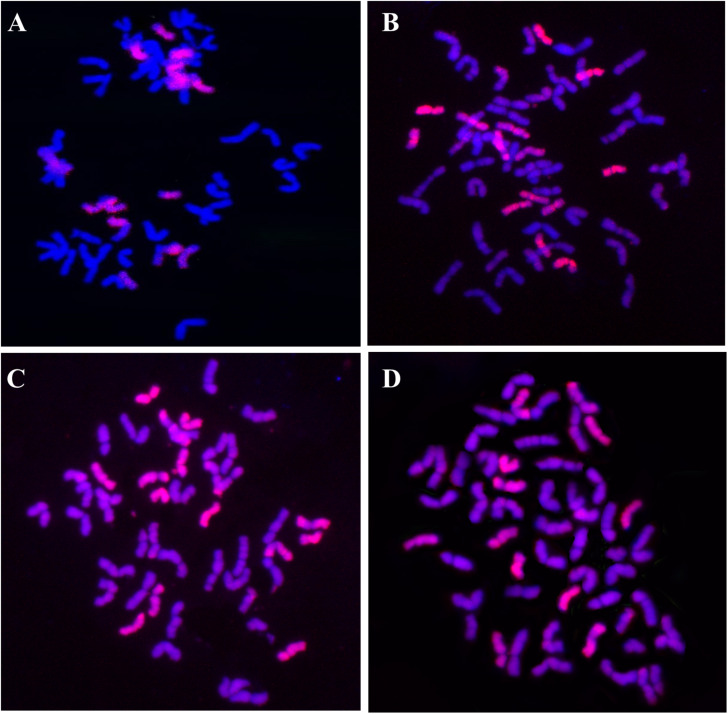
GISH patterns of root-tip cells of the four perennial wheat lines probed with the *Th. intermedium* genomic DNA and blocked with the Chinese Spring genomic DNA. The *Th. intermedium* and wheat chromosomes showed lilac-pink color and blue color, respectively. **(A)** SX12-787, 2n = 56, 14 *Th. intermedium* chromosomes and 42 wheat chromosomes. **(B)** SX12-1150, 2n = 56, 14 *Th. intermedium* chromosomes and 42 wheat chromosomes. **(C)** SX12-480, 2n = 48, 16 *Th. intermedium* chromosomes and 32 wheat chromosomes. **(D)** SX12-1269, 2n = 54, 12 *Th. intermedium* chromosomes and 42 wheat chromosomes.

GISH analysis was applied using the *Th. intermedium* total genomic DNA as a probe and the Chinese Spring total genomic DNA as a blocker to detect the *Th. intermedium* chromosomes in the perennial wheat lines. Lines SX12-787 and SX12-1150 had a mitotic chromosome number of 2n = 56. Fourteen chromosomes showed lilac-pink fluorescence signals, and 42 chromosomes were counterstained with blue fluorescence signals, indicating that both SX12-787 and SX12-1150 contained 14 *Th. intermedium* chromosomes and 42 wheat chromosomes (Figures 7A,B). Lines SX12-480 and SX12-1269 contained 48 and 54 chromosomes, respectively. Based on the fluorescence patterns on chromosomes, line SX12-480 (2n = 48) had 16 chromosomes originated from *Th. intermedium* and the other 32 chromosomes belonged to wheat (Figure 7C). In line SX12-1269 (2n = 54), there were 12 *Th. intermedium* chromosomes and 42 wheat chromosomes (Figure 7D).

## Discussion

Four dual-use perennial lines were developed, which displayed persistent regrowth capability for at least 3 years. Post-harvest regrowth habit allowed to test the hypothesis that perennial grain yield potential declines with increase of plant age. The perennial wheat lines exhibited promising resistance against the parasite cyst nematode *H. avenae* and *H. filipjevi*. These lines were characterized by their superior amino acid contents, and other nutrient compositions such as crude protein, crude fat, P, and Se for both forage and grain over the common wheat control.

The agronomic potential of perennial wheat lines was studied over 3 years, as grain yield may decrease over plant age. Plant height and the grain yield components (i.e., number of spikelets per spike for line SX12-480 and thousand-kernel weight for line SX12-787) for the 3-year-old perennial genotypes relative to one- or 2-year-old genotypes were lower in general. However, spike length and the yield components (i.e., number of spikelets per spike and thousand-kernel weight) remained consistent over time in the perennial wheat lines SX12-1150 and SX12-1169. In many cases, yield declines have been observed in perennial cereals (Tautges et al., 2018), although a number of perennial genotypes showed the consistency in yield components over several years (Jaikumar et al., 2012), or even increases in grain yield (Hayes et al., 2012). The decline of grain yields in 2-year-old plants reflected plant age effect on yield components (Jaikumar et al., 2012). Fundamentally, grain yield penalty over plant ages in perennial plants is associated with trade-off between reproduction and post-harvest regrowth. Perennial plants allocate more resources below ground for extending life span even under harsh environmental conditions and less to seed/grain production (Jaikumar et al., 2012; Vico et al., 2016). Combining consistent grain yield potential with persistent post-harvest regrowth habit may be challenging in perennial wheat breeding programs. Instead, breeding efforts could focus on increasing forage biomass production in order to offset the age-related decline in grain yield.

The generally lower yield potential of the 3-year-old plants was not surprising given the other important fact as the accumulation of (root) pathogens, such as foot rot [caused by *F. culmorum* (W.G. Smith) Sacc.], crown rot (caused by *F. pseudograminearum* O’Donnell & T. Aoki), common root rot [caused by *Cochliobolus sativus* (Ito & Kuribayashi) Drechs. ex Dastur], and damping-off/root rot (caused by *Pythium* spp.) (Bell et al., 2010; Jaikumar et al., 2012). The epidemic of plant disease in annual crops always dies out after a few months, till host plant harvest/die; however, this is not the case for the perennial plants. After harvest, plant residues left on the soil surface may result in increase of soil moisture and decrease of soil temperatures, which promote growth of soilborne pathogens (Bockus and Shroyer, 1998). Cox et al. (2005) found that perennial wheat lines were highly susceptible to take-all (caused by *Gaeumannomyces graminis* var. *tritici* J. Walker), resulting in large amount of biomass loss. Little information has been available on the resistance to common root pathogens and pests in perennial donors, as well as their derivatives. So far, only few screening has been publicly available on the phenotypic responses of wheat-*Thinopyrum* hybrids to CCN (Li et al., 2012). The wheat-*T. intermedium* derivatives were found to be the most resistant group of lines against *H. filipjevi* among the germplasm tested in that study including wheat-*Th. ponticum* derivatives and common wheat cultivars. It should be noted that the perennial wheat-*Th. intermedium* lines in our study displayed consistent resistance to both CCN species *H. avenae* and *H. filipjevi*, which could suppress the multiplication of CCN in the soil.

During the last decades, the mineral and protein concentrations of annual wheat cultivars have shown a continuous decline coinciding with the significantly increased grain yield (Fan et al., 2008). Common wheat cultivars are known to be low in content of some essential amino acids, especially lysine and threonine, the two most deficient amino acids (Abdel-Aal and Sosulski, 2001; Horvatić and Ereš, 2002). An effective method to counteract micronutrient malnutrition is to exploit the genetic variation of overall micronutrient availability in the wild wheat relatives and develop cultivars with enhanced nutritional values (Monasterio and Graham, 2000; Murphy et al., 2009). Wild relatives of wheat, such as *Th. intermedium* and *Th. elongatum* (Host) D. R. Dewey, are well-known for their beneficial characteristics including improved nutritional values (Murphy et al., 2009; Tyl and Ismail, 2019). The wide adaptation of *Th. intermedium* and *Th. elongatum* in hybrid breeding was contributed by their superior end-use quality and nutritional profile. Partial amphiploids derived from different *Thinopyrum* species were found to have higher levels of carotenoids, soluble polyphenols, alkilresorcinols, and better gluten digestibility than common wheat (Gazza et al., 2016). *Thinopyrum intermedium* seed was reported to have a higher concentration of protein and almost all essential amino acids than wheat (Marti et al., 2015). The superiority of *Th. intermedium* over common wheat was highlighted in terms of protein and fiber contents (Marti et al., 2015). Notably, mineral nutrient concentrations and chemical compositions in the newly developed perennial wheat lines were better than the annual cultivar Jinchun 9 for crude protein, crude fat, P, and Se. The perennial wheat lines, in particular, SX12-787 and SX12-1269, were superior in the improvement of wheat nutritive level.

Perennial growth habit is a quantitative trait and is mediated by multiple genes on different alien chromosomes (Cox et al., 2006; Dehaan et al., 2014). The genetic control is not fully exploited yet and one of the loci involved in this post-harvest regrowth capacity is mapped on chromosome 4E of *Th. elongatum* (Lammer et al., 2004). It is particularly worthy of mention that lines SX12-480, SX12-1150, and SX12-1269 persisted to post-harvest regrowth over three to five successive years in the field (unpublished data). GISH patterns showed the presence of 12-16 intact alien chromosomes in the perennial lines. The amplification profiles of the markers specified alien introgression of chromosomes 2ES, 3EL, 7ES, and 7EL in the four perennial wheat lines. Although speculating, whether the perennial regrowth habit is linked to these alien introgression remains to be determined. The first consensus genetic map of *Th. intermedium* has already been available (Kantarski et al., 2017), which provides a valuable tool for wheat breeders to map CCN-resistant gene(s) and the key players involved in the post-harvest regrowth trait.

The perennial wheat generally has unique morphological traits that are quite divergent from annual wheat. Usually, the wild perennial species have performed poorly for some domestication traits, such as small spike and seed size, which need to be rectified in perennial wheat varieties (Davies and Hillman, 1992; Kantar et al., 2016). Common wheat cultivars have a range of thousand-kernel weight from 35.2 to 40.3 g, while approximate 5.9–7.8 g is found for the *Th. intermedium* entries (Larkin et al., 2014). Thousand-kernel weight of the perennial wheat line SX12-787 was as high as 25.5 g in the first year and the other perennial wheat lines yielded a range from 12.1 to 17.3 g (Figure 4). The increase in grain weight is probably attributed to the selection criteria in the early generations in which intermediate type of the F_2_ and F_3_ derivatives were selected for advancing to next generations (Figures 2A,B). However, the second year thousand-kernel weight varied from that of the first year, which was consistent with previous research by Hayes et al. (2018). Further research is needed to investigate the relationship of grain yields among the 3 years of harvest and develop better adapted perennial wheat that combined the grain yield and perennial habit. Besides, the four perennial wheat lines were tall in plant height (118.3–146.7 cm). This trait is known to closely relate to forage biomass (Trotter and Frazier, 2008).

## Conclusion

We have developed four perennial wheat-*Th. intermedium* lines, which showed the remarkable capability to regenerate and persist at least 3 years in the field. Promising resistance was identified to both species of *H. avenae* and *H. filipjevi* that are prevalent in China in these lines. They had superior amino acids and other nutrient compositions for forage and grain over the common wheat control. Analyses of *Thinopyrum* genome-specific molecular marker and GISH indicated that these lines had 12–16 *Thinopyrum* chromosomes. Currently, these perennial wheat lines have been evaluating at multiple geographic locations to test their adaptability to different harsh environments. Meanwhile, new perennial wheat lines are being made using these lines as parents. The development of perennial species has been proposed as an approach to improve the sustainability of agriculture, conserving natural resources while producing food and forage.

## Data Availability Statement

The datasets generated for this study are included in the article or are available on request to the corresponding authors.

## Author Contributions

LC and YS conceived and designed the research. LC, YR, YZ, QG, ZT, YN, WY, and YS conducted the experiments and analyzed the data. LC and HL wrote the manuscript. All authors contributed to the article and approved the submitted version.

## Conflict of Interest

The authors declare that the research was conducted in the absence of any commercial or financial relationships that could be construed as a potential conflict of interest.

## Footnotes

1 https://data.cma.cn/en
